# [Corrigendum] FHOD1 is upregulated in gastric cancer and promotes the proliferation and invasion of gastric cancer cells

**DOI:** 10.3892/ol.2026.15776

**Published:** 2026-07-21

**Authors:** Chengfei Jiang, Binbin Yuan, Bo Hang, Jian-Hua Mao, Xiaoping Zou, Pin Wang

Oncol Lett 22: 712, 2021; DOI: 10.3892/ol.2021.12973

Following the publication of the above paper [and an Expression of Concern statement (doi: 10.3892/ol.2026.15632) that was published after it had been drawn to the Editor's attention by a concerned reader that, regarding the cell invasion assay data shown in Fig. 8A on p. 7, two pairs of data panels shown for the shFHOD1 and LV-FHOD1 experiments contained overlapping sections], the authors have responded to the Editorial Office to address this issue. (A subsequent investigation of the data in this paper undertaken by the Editorial Office also revealed that a pair of the Control data panels in Fig. 8B also contained an overlapping section of data).

The authors wish to explain that the data panels in Fig. 8A and B were intended to show representative microscopic fields from the same treatment groups, although certain of the fields of view were found to be mutually overlapping. Even though the data were assembled in this figure intentionally as shown, to avoid any possible further confusion the authors have agreed to publish a revised version of Fig. 8 (which is featured on the next page), where the data shown within each of the data panels is now entirely discrete from the others.

The authors are grateful to the Editor of *Oncology Letters* for allowing them this opportunity to publish a Corrigendum, and all the authors agree with its publication. The authors also thank the reader for drawing this matter to their attention.

## Figures and Tables

**Figure 8.**
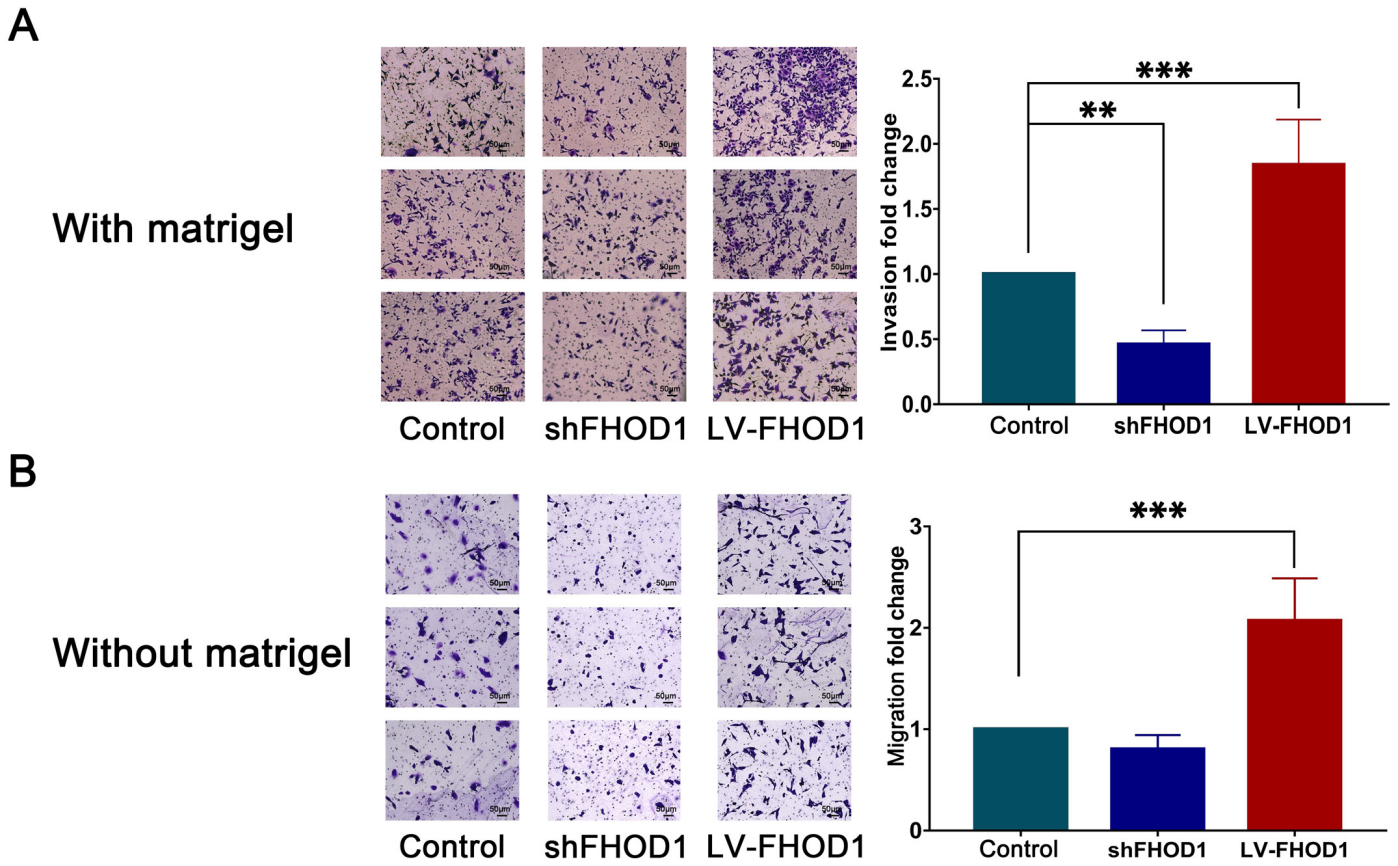
Invasive ability of GC cells after knockdown and overexpression of FHOD1 with or without Matrigel. (A) Left: The number of HGC-27 cells crossing the basement membrane with Matrigel (magnification, ×100). Right: Quantification of GC cells crossing the basement membrane after knockdown and overexpression of FHOD1. (B) Left: The number of HGC-27 cells crossing the basement membrane without Matrigel (magnification, ×100). Right: Quantification of GC cells crossing the basement membrane after knockdown and overexpression of FHOD1. **P<0.01 and ***P<0.001. FHOD1, formin homology 2 domain containing 1; GC, gastric cancer; sh, short hairpin; LV, lentivirus.

